# Supplementary material for: Familial 4p Interstitial Deletion Provides New Insights and Candidate Genes Underlying This Rare Condition
Source: Genes (Basel). 2023 Mar 3;14(3):635. doi: 10.3390/genes14030635 (PMC10048360; doi:10.3390/genes14030635)
Supplement: Supplementary file 1 [file genes-14-00635-s001.zip › genes-2257321-supplementary.pdf]

Supplemental

Table S1. Phenotypic features of reported interstitial 4p deletion cases during the past 45 years

References		***Bands***	***General***			***Manifestation***												***Development***		
	Case	Deleted region	Age	Sex	Race	a	b	c	d	e	f	g	h	i	j	k	l	m	n	o
This study	Index	14-15.2	1y	F	W															
	2				W															
Francke et al., 1977	3	11-15.2	30y	F		+	-	-	-	+		+	-	+	+	+	+	-		
Nielson et al., 1977	4	12-15					+	+	+	+			+			+	+	+		
Ray et al., 1984[10]	5	14-15.32				-	+	+	+	+	+	-	-	-	+		+	-		
Wang et al., 1986[14]	6	14-15.32																		
Ishikawa et al., 1990[14]	7	15.2-15.32				-	+	-	-	-	+	+	+	+			+	-		
Fryns et al.,[15] 1989/1995	8	14-15.3	6y	M	W	+	+	+	+	-	+	-	-	+	-	+	+	+	-	+
Davies et al., 1990	9	15.3																		
Romain et al., 1985	10	15.2-15.33		F		-	-	-	-	-	-	-	-	-	-	//	+	+	-	n/a
White et al.,1995[5]	11	14-16.1	14y	M	W	+	+	+	+	+	+	+	+	+	+	+	+	+	+	+
	12	15.2-16.1	5m	F	H	+	+	+	+	-	+	+	+	+	+	+	+	+	+	
	17	14-15.32	10y	F	A	-	+	+	+	-	-	+	-	+	-	+	+	+		
Chitayat et al., 1995	18	15.2-15.33	10y	M	W	+	+	-	-	+	+	+	-	+	+	+	+	-	-	+
	19	15.2-15.33	11y	F	W	+	+	+	-	+	-	+	-	+	-	+	+	+	//	n/a
	20	14-15.2	25y	M	W	+	+	//	+	-	-	+	+	//	+	-	+	-	+	+
Gawlik-Kuklinska et al., 2008	21	14-15.32	4y	M		+	+	+	+	+	+	+	+	+	+	+	+	+	+	-
Bailey et al., 2010	22	14-15.33	2y	F	W	-	-	+	+	+	-	-	-	-	-	-	+	+	+	n/a
Chen et al., 2013[43]	23	14-15.2	Prenatal									n/a								
Su et al., 2011[8]	24	12-15.2	7y	F	A	+	+	+	+	+	+			+			+			
Alesi et al., 2011[2]	25	15.31-15.32	14m	M	W	-	-	+	-	+	-	-	+	-	-	+	-	-	-	-
Tonk et al., 2003[7]	26	15.2-16.1		F		+	+	-	+	+	-	-	-	-	-	-	+	-	-	
Estabrooks et al., 1995	27	15.31-16.3		M		-	//	-	-	+	-	-	-	-	-	-	+	+	-	-
Moller et al., 2007[12]	28	15.1-15.31	38y	F	W	+	+	-	-	+	+	-	-	-	-	-	+	-	-	n/a

Piccione et al.,2015	29	15.32-16.1 dup	3y	F	A	-	-	-	-	-	-	-	-	-	-	-	+	+	+	n/a
Parks et al., 2020[17]	30	15.1-15.31	5y	M	A	-	-	-	-	-	-	-	-	-	-	-	+	-	+	+
Mitroi et al., 2017[13]	31	15.1-15.31	3y	M	W	+	+	-	-	+	+	+	-	-	+	+	+	-	-	+
Wu LQ et al., 2008	34	15.2-16.1	24y	F	A	-	-	-	+	+	-	+	-	-	-	+	+	-	-	n/a
Innes A et al.,2001	35	15.2-16.1	3y	M		-	+	-	-	-	+	-	-	-	-	+	+	-	-	-
Volleth M et al., 1993	36	15.2-16	7y	F																
de Graaf GV et al., 1997[6]	37	12-15.1	7y	F		+	-	+	-	-	-	-	-	-	-	-	+	-	-	n/a

a) Long face
b) Mid face hypoplasia
c) Upslanted fissures
d) Epicanthal folds
e) Large beaked nose
f) Thick lower lip
g) High palate
h) Micrognathia
i) Broad/short neck
j) Broad hands/feet
k) Tall/thin body
l) Delay/retardation
m) Hypotonia
n) Congenital heart disease
o) Cryptorchidism
